# Benefits of Temporally-Resolved, Policy-Relevant, Data-Informed Technoeconomic Evaluation of Multifunctional Systems: H_2_ Deployment in a District Energy System

**DOI:** 10.1021/acssuschemeng.5c07425

**Published:** 2026-04-23

**Authors:** Diego A. Hincapie-Ossa, Chris Swanson, Daniel B. Gingerich

**Affiliations:** † Department of Civil, Environmental and Geodetic Engineering, 2647 The Ohio State University, Columbus, Ohio 43210, United States; ‡ Engie North America, Columbus, Ohio 43210, United States; § Department of Integrated Systems Engineering, The Ohio State University, Columbus, Ohio 43210, United States; ∥ Environmental Science Graduate Program, The Ohio State University, Columbus, Ohio 43210, United States; ⊥ The Sustainability Institute, The Ohio State University, Columbus, Ohio 43210, United States

**Keywords:** polygeneration, multifunctional systems, technoeconomic assessment, low-carbon hydrogen, solid-oxide electrolyzer

## Abstract

Hydrogen (H_2_) production and usage can be integrated into existing infrastructure to reduce carbon emissions and increase fuel supply security. In this work, we design and execute a technoeconomic analysis for a multifunctional District Energy System (DES) that produces and uses H_2_. We parametrically analyze levelized costs for H_2_, oxygen, and carbon dioxide abatement and capture to evaluate the viability of integrating H_2_ production, using Solid Oxide Electrolysis Cells, for a DES that could alternatively purchase H_2_ and install carbon capture utilization and storage (CCUS). We find that different project alternatives are distinctively sensitive to certain product prices, and therefore suitable for particular markets. Self-production alternatives with large H_2_ production do not require carbon pricing, so long as oxygen revenues are high. Conversely, when H_2_ is purchased, carbon prices in excess of $209/Tonne_CO2e_ are required for project viability. Projects with CCUS that purchase H_2_ depend on carbon-related revenues–even when hydrogen is free. When purchasing H_2_ in scenarios without carbon pricing, only projects using renewable-energy-based H_2_ and no CCUS implementation are viable for nonzero H_2_ prices (<$0.85/kg_H2_). Our work demonstrates that evaluating the levelized costs of all products in combination is necessary to assess the economic feasibility of multifunctional systems.

## Graphical abstract

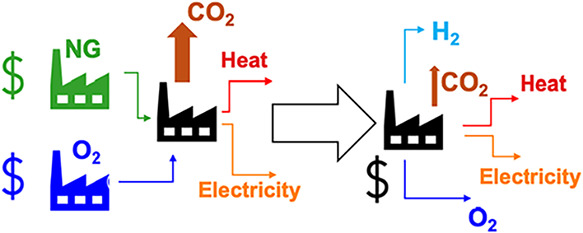

## Introduction

Industrial transformations seeking efficiency and improved economic and environmental performance often involve risks for current energy producers and users. For industrial decision-makers, strategies that diversify operations (e.g., dual fuel conversion, process integration, or implementing multifunctional systems) can leverage potential economies of scale, derisk their business, and improve a system’s environmental performance^1^ and mass and energy efficiencies.^2–4^ Dual-fuel power plants benefit from additional flexibility^5,6^ and combined heat and power plants (CHP)^7^ offer higher efficiencies relative to separately producing heat and electricity (65–75% energy efficiency vs ∼50% for separate processes).^8^

Diversification strategies, implemented as multifunctional systems (MFSs) can also support utility owners in guiding district energy systems (DES) through the energy transition.^9,10^ These solutions are expected to become common as coal generating capacity retires at an accelerated rate.^11^ Institutional facilities (e.g., hospitals and universities^8^) that also demand commodities and utilities such as fuels, steam, and oxygen (O_2_) are increasingly adopting DES architectures. Fuel diversification is emerging as a possible transition pathway toward more secure operation.

Integrating production and usage of hydrogen by retrofitting reconfigured DESs can support the development of more secure and diverse energy systems^12–14^ with reduced environmental impacts,^12,15–18^ including lower greenhouse gas emissions. Different feedstocks and processes can be used to produce hydrogen fuel^12,19^ – including water electrolysis – for use in industrial processes without use-phase CO_2_ emissions.^20,21^ However, climate benefits depend on the production method^22,23^ and trade-offs exist between climate and economic effects.^20^ Natural gas steam reformation is the lowest-cost production technology – with a levelized cost of hydrogen (LCOH) of $1.06/kg.^24^ Electrolysis powered with renewable electricity (green H_2_) and from fossil fuels with carbon capture, utilization, and storage (CCUS) (blue H_2_) are more expensive.^20,25^ Green H_2_ highly depends on electricity costs varying from $3.5–9.6/kg_H2_;^20^ gasification with CCUS, $2.7–3.7/kg_H2_;^20^ and steam reforming with CCUS, $1.3–2.3/kg_H2_.^24^ Therefore, low-carbon H_2_ will likely need policy support (e.g., Important Projects of Common European Interest^12,26^ ) to compete with steam reformation while associated processes mature and learning-by-doing occurs.^12,19^

Solid Oxide Electrolyzer Cells (SOEC)^14,27^ are emerging as a promising^28^ production option. This electrolysis process operates at higher temperatures and can offer higher system-level efficiencies relative to more mature technologies like Proton Membranes (PM) and Alkaline Water Electrolyzers (AEM)–requiring 45–55 vs 57–69 and 50–83 kWh/kg_H2,LHV_, respectively, at bench-scale.^28^ This increased efficiency is mainly because a portion of electrical energy can be replaced with thermal energy in steam,^28–30^ making it suitable for integration with steam-producing processes.^14,31,32^ Additionally, SOEC technology and cost improvements are likely to be greater than those expected for PM and AEM.^27,28^ However, current SOEC deployment is limited with only one >1 MW operating project in 2023.^33^ Therefore, there is large uncertainty for SOEC system components (e.g., electrolyzer stacks) and deployment at utility-system scales.^34^

Technoeconomic analysis (TEA) is a well-established method to evaluate technology costs, including emerging ones;^35^ however, actual operation can significantly differ from assumed design operation conditions^36^ challenging early stage TEA’s ability to evaluate viability.^37^ When evaluating technology integration into existing facilities, this can be overcome with primary data (e.g., actual commodity price structures, asset configuration, and operation rules) from operators. For generation systems, using actual demand data allows for tailored models that incorporate equipment efficiency curves. Furthermore, TEAs commonly use fixed parameters to model efficiencies, as exemplified in the extant TEA literature (and best practices)^38^ and in reported studies for emerging H_2_ technologies.^37,39^ The use of efficiency curves in TEAs that are supported by real data can identify patterns at actual operational resolutions (e.g., hourly) that models with coarser or fixed efficiency assumptions cannot reflect.

The analysis of dependent products and their interactions in MFS, including H_2_ ones, has been understudied in the TEA literature. In fact, although cost allocation is a common–and critical–problem in coproduction, it is rarely addressed in the broader TEA literature.^40^ In recent work, Ktori et al.^41^ conclude that traditional economic evaluation of MFS with nonallocation methods tends to overestimate costs of the determining products. In this thrust of work, the authors allocate costs to multiple products using economic allocation methods based on life-cycle assessment approaches–and find substantial differences with nonallocation methods.^42,43^ In the H_2_ literature, while studies of coproduction or polygeneration identify cost reductions resulting from adopting MFS architectures,^1,44^ these seldom include price allocation or interactions between product costs and associated uncertainties. One common assumption is to fix the price of products that are considered less uncertain before calculate a LCOH.^45–53^ Another approach is to determine levelized costs under a specific scenario or optimal conditions^54–56^ or frame multifunctional analysis as a sensitivity analyses.^57,58^ A selection of TEAs on MFSs and trends in the literature in Supporting Information (SI) Section 1 and Table S1.

In this work, we codevelop with a DES system operator a TEA to assess MFS economic feasibility that is parametrized with actual plant operation data. With this approach, we evaluate a district reconfiguration to integrate an SOEC system to produce H_2_ for self-consumption, as an alternative to natural gas. We evaluate a large set of project designs, operation alternatives, and associated uncertainties (e.g., fuel and electricity prices or interannual weather variations) via parametric approaches. This allows us to determine the levelized costs of multiple products and the feasibility price ranges for combinations of H_2_, O_2_, and CO_2_ capture or abatement revenues. We further contribute to the TEA literature by quantifying the added value of incorporating high-resolution DES operational data and demonstrating the benefits of systematically evaluating the levelized cost of multiple products in MFSs.

## Methods

We assess the economic viability of implementing H_2_ production and usage in a DES by quantifying changes in financial performance after reconfiguring the district to integrate an SOEC. We developed a model representing one year of DES operation at hourly resolution (the 8760 module) using actual data from a DES and calculated the resulting annual cash flow changes. With these changes, we determined the levelized costs of three products (the life-cycle module): H_2_, O_2_, and CO_2_ abatement (Figure 1A). Additionally, we assessed projects with CCUS, adding captured CO_2_ as a fourth product. Using parametric analysis, we fix the price of three products to calculate the levelized cost of the fourth. By repeating this, we generate curves that define viability regions for price combinations of two products. We complement this with sensitivity analyses to evaluate the impact of things exogenous to the DES operator (e.g., commodity prices and weather).

**Figure 1:**
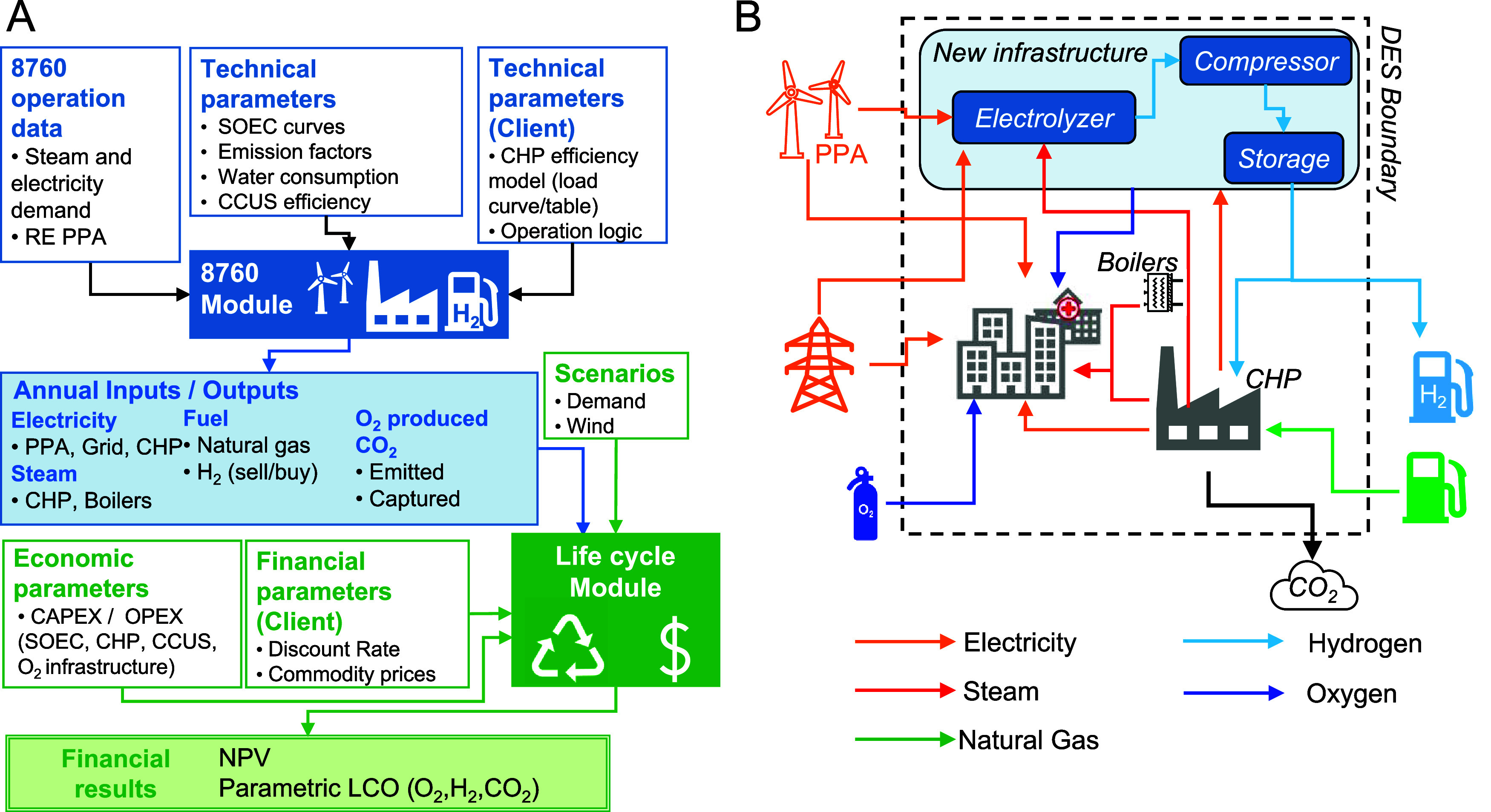
Model and System Description. Panel (A) shows the methods map comprising the 8760 and life-cycle financial module. Panel (B) represents the general DES arrangement and the energy and commodities’ flow in system. A SOEC system project demands electricity and heat to generate H_2_ and O_2_. A DES system diagram and its operational rules are presented in SI Section 2 and in Figure S2. Image modified from ref 59.

### Operation Baseline

We use 2022 data for wind production and energy demand to define the DES operational baseline. This baseline includes multiple energy (thermal and electrical) sources to meet the demands of an educational campus with academic and administrative buildings, a medical complex, student residences, and sports facilities. The main energy source is a central combined-cycle CHP plant with two combustion turbines and one steam turbine for an overall capacity of 100.4 MW. The CHP is complemented by industrial boilers and external electricity sources to cover unmet demand (Figure 1B). In the baseline, the central plant and the boilers use externally supplied natural gas. In addition, the DES is served by a Power Purchase Agreement (PPA) from a wind farm and is connected to the regional electricity grid. Finally, the medical complex requires O_2_ presently supplied by an external vendor.

#### DES Service Costs

Our codeveloped model uses the CHP operator’s rates and cost data and considers a variety of electrical energy and capacity charges. The electricity services’ cost, *C*_E_ (in [$/yr]), is the sum of two electricity and two power-related charges described in eq 1.

1 $$C_{E}=E_{\mathrm{PPA}}K_{\mathrm{PPA}}+E_{\mathrm{grid}}K_{\mathrm{grid}}+C_{\mathrm{trans}}+C_{\mathrm{cap}}$$

The electricity components include PPA (*E*_PPA_ *)* and retail (*E*_grid_ *)* demand (in [MWh]) paid at prices of *K*_PPA_ (of $50/MWh) and *K*_grid_ (of $43/MWh), reported by the operator for actual conditions. The wind PPA has a “pay-as-produced” structure with a maximum capacity (*E*_PPA,max_, in [MW]) of 50 MW that supplies available wind-powered electricity, denoted *E*_W_ (in [MWh]). The grid provides reliability services and the district pays a fixed amount for transmission charges, *C*_trans_ (in [$]), and a capacity costs *C*_cap_ (in [$]) to support this. The former depends on monthly peaks and the latter on total electricity demand during five peak hours in the grid region (SI Section 3). In the baseline, the main costs of DES-produced energy are natural gas (NG) purchases, the cost for which (eq 2) includes purchases for the CHP and boiler (*F*_CHP_ and *F*_B_, in [MMBTU]) purchased at a fixed price, *K*_NG_ (of $6.00/MMBTU).

2 $$C_{\mathrm{NG}}=\left({F_{\mathrm{CHP}}+F}_{B}\right)K_{\mathrm{NG}}$$

The operational logic of energy supply seeks to minimize cost, given physical capacity limits of different units. The wind PPA is the first source to meet electricity demand, denoted *D*_E_ (in [MWh]) (eq 3). When the available wind or the limit *E*_PPA,max_ is reached, the CHP then operates within operational bounds. The CHP operational bounds define the hourly energy limits of our 8760 model, denoted as *E*_CHP,min_ (16 MWh) and *E*_CHP,max_ (104.3 MWh) as described in eq 4. While this is a substantial range in capacity, the DES load served by this CHP is highly variable. Given this, the turbomachinery in the facility was specifically selected to operate over this range. If the loads lie below the lowest output in the operational range (one combustion turbine operating at its maximum turndown), the CHP does not operate and instead, the grid supplies the remaining electricity demand, *E*_grid_ (eq 5).

3 $$E_{\mathrm{PPA}}=\min\left(E_{\mathrm{PPA},\max},E_{W},D_{E}\right)$$

4 $$E_{CHP}=\{\begin{array}{l} 0,\mathrm{for},D_{E}-E_{PPA}<E_{CHP,\min}\\ \min\left(D_{E}-E_{PPA},E_{CHP,\max}\right),\mathrm{for},D_{E}-E_{PPA}\geq E_{CHP,\min} \end{array}$$

5 $$E_{\mathrm{grid}}=D_{E}-E_{\mathrm{PPA}}-E_{\mathrm{CHP}}$$

The first source of heat to meet the demand, *D*_Th_ (in [MMBTU/h]), is the CHP denoted *Q*_CHP_ (in [MMBTU]), until reaching its maximum production *Q*_CHP,max_^*t*^ (in [MMBTU]). This maximum depends on the instantaneous electricity production of the CHP (eq 6). Additional heat needs, *Q*_B_ (in [MMBTU]), are supplied by the boilers (eq 7). The maximum DES heat supply is 500,000 lb/h (561.5 MMBTU/h, calculated at 1123 BTU/lb, the enthalpy of the steam circuit) and corresponds to the sum of *Q*_CHP,max_^*t*^ and the boiler capacity maximum, *Q*_B,max_. DES model parameters are listed in SI Section 5 and Table S2.

6 $$Q_{\mathrm{CHP}}=\max\left(D_{\mathrm{Th}},Q_{\mathrm{CHP},\max}^{t}\right)\;\mathrm{with}\;Q_{\mathrm{CHP},\max}^{t}=f\left(E_{\mathrm{CHP}}\right)$$

7 $$Q_{B}={\min(D}_{\mathrm{Th}-}Q_{\mathrm{CHP}},Q_{B,\max})$$

#### Technical Data and Efficiency Models

We model hourly efficiency as a function of operating conditions, differentiating us from other TEAs that use fixed parameters to characterize H_2_ production^39^ or power^38,60^ system efficiencies. Using CHP’s electricity and thermal loads, we use the second-law efficiency to calculate the resulting fuel consumption, *F*_CHP_ (in [MMBTU]) as shown in eq 8. To determine the efficiency, η_CHP_, under any operating condition, we use a performance curve of the CHP. This performance surface was created by the DES operator in software that models turbomachinery operation. As a sensitivity analysis, we also model the system under the assumption of fixed CHP and electrolyzer efficiencies to quantify the impact of using a fixed efficiency. We show a simplified representation of the performance surface in SI Section 4 and Figure S3. Since boiler efficiency does not vary greatly, their fuel consumption is calculated using a fixed average efficiency value of 0.80, reported by the operator (eq 9).

8 $$F_{\mathrm{CHP}}=\frac{{(Q}_{\mathrm{CHP}}+{3.412E}_{\mathrm{CHP}})}{\eta_{\mathrm{CHP}}}$$

9 $$F_{B}=\frac{Q_{B}}{\eta_{B}}$$

When primary data were unavailable, we used operator references and estimates and secondary sources. We calculate the O_2_ expenses, *C*_O2_ (in [$/kg_O2_]) using DES’s medical complex consumption (in [kg/h]), and a $0.132/kg O_2_ price. Similarly, we estimate the water consumption cost, *C*_H2O_ (in [kg/h]), using water use costs of $3.72/Tonne. Environmental impact factors (e.g., carbon emissions, water use) were provided by the operator. We determine the net present value (NPV, eq 10) for the costs, represented as *C* (in [$]) with subindices denoting each cost item. We determined nonfuel variable costs for the central plant, *C*_CHP,Var_ (in [$/MWh]), from the Job and Economic Development Impact tool^61^ and GHG emissions intensity factors for gas-fired CHPs from the Federal LCA Commons.^62,63^ We use those intensity factors to calculate the system emissions to evaluate carbon costs when GHG are priced [$/Tonne_CO2e_]. A list of technical and cost parameters for the evaluated alternatives is presented in SI Section 5 and Table S2, including parameters to calculate the costs associated with water withdrawal, oxygen consumed, and carbon emissions (*C*_H_2_O_, *C*_O_2__, and *C*_CO_2e,emmitted__, respectively, in [$]). Fixed costs are not included, since they do not vary after project implementation. We calculate the NPV (in [$]) over *N* years of project life, using the DES operator interest rate, *i* (6%/yr).

10 $$C_{\mathrm{NPV}}^{\mathrm{base}}=\sum_{n=1}^{N}{(C}_{E}+{{C_{\mathrm{NG}}+C}_{\mathrm{CHP},\mathrm{Var}}+C}_{H_{2}O}+C_{O_{2}}+C_{CO_{2e},\mathrm{emitted}})\frac{1}{{\left(1+i\right)}^{n}}$$

### Technoeconomic Alternatives

#### Technology Alternatives

An SOEC can be connected to the district’s heating circuit to produce H_2_ from 150 and 650 °C steam. With published efficiency curves^64^ for an SOEC operating at 5000 A/m^2^ to calculate the required energy, Δ*H*, (in [MMBTU]), using as parameters the feedwater and electrolysis temperatures. The Gibbs free energy, denoted Δ*G* (in [MMBTU]), sets the share of process energy consumed, *E*_El_, (in [MWh]) that corresponds to electricity (eq 11), which varies with process temperature. The remainder corresponds to thermal energy, *Q*_El_, (in [MMBTU]), about 20% of the electrolyzer’s total energy consuption.^64^ A heat exchanger recovers heat from the oxygen and hydrogen streams for inlet preheating, reducing heat and electricity demands. The remaining demands are met by the DES energy sources.

11 $$\Delta H=\Delta G-T\Delta S={3.412E}_{\mathrm{El}}+Q_{\mathrm{El}}$$

In addition to the SOEC, we also analyze CCUS implementation alternatives. We develop the technoeconomic model for the two systems (eq 12) using literature data. We use the midpoint SOEC capital expenditure (CAPEX) estimate for electrolyzers with 5–10 MW capacities ($ 2541 kW_El,in_).^27^ We define operational expenses (OPEX) (except from electricity), *C*_SOEC,VAR_ (in [$/MW]), are 3% of the capital costs, a common assumption in the literature.^65,66^ For CCUS operation, we use a capture rate of 85% ^67–70^ and energy penalty of 25% ^68,71,72^ of nominal CHP power output, an additional electricity load to the DES, met by the grid. We considered $19.43/Tonne for the CCUS OPEX corresponding to CO_2_ compression, transportation and storage (*C*_OPEX,CCUS_ (in [$])).^24^ The system also generates revenue from excess H_2_, *R*_H2_, and by selling captured CO_2_, *R*_CO2,capt_ (both in [$]).

12 $$C_{\mathrm{NPV}}^{\mathrm{Proj}}=\sum_{n=0}^{N}(C_{\mathrm{CAPEX},\mathrm{SOEC}}+C_{\mathrm{CAPEX},\mathrm{CCUS}}+C_{E}+{{C_{\mathrm{NG}}-R_{H_{2}}+C}_{\mathrm{CHP},\mathrm{Var}}+C_{\mathrm{SOEC},\mathrm{VAR}}+C}_{H_{2}O}+C_{O_{2}}+C_{CO_{2},\mathrm{emitted}}+C_{\mathrm{OPEX},\mathrm{CCUS}}-R_{CO_{2,\mathrm{capt}}})\frac{1}{{\left(1+i\right)}^{n}}$$

We assess projects with different SOEC size and economies of scale. With a base capacity of 5MW_NET‑INPUT_ for the SOEC, we calculate the sum of the SOEC CAPEX, escalating each electrolyzer component *M* to size *X* (in [MW_In_]) using dimensionless factor *S*.^27,28^ We use technoeconomic values reported by Böhm et al.^27^ (reported SI Section 6 and Table S3). The CCUS has a fixed capacity set by the CHP size, so we do not escalate its size or cost. The CCUS cost was calculated as 76%^73^ of the reference cost of the power plant as described in SI Section 5 and Table S2.

13 $$C_{\mathrm{CAPEX},\mathrm{SOEC},X\mathrm{MW}}=\sum {C_{\mathrm{CAPEX},\mathrm{SOEC},5\mathrm{MW}}\left(\frac{X\mathrm{MW}}{5\mathrm{MW}}\right)}^{S_{M}}$$

#### Business Model Alternatives

We evaluate a **base-design** project (defined in the SI, Section 5 and Table S2) under the following operation strategies.

- **Alternative 0** (**Baseline)**: The DES operates without H_2_ and CCUS and purchases natural gas and O_2_ from external vendors.
- **Alternative 1 (SOEC project with High-integration (Hi-SOEC))**: The DES has an installed SOEC and maximizes H_2_ production for CHP use, partially substituting and reducing dependence on natural gas.
- **Alternative 2 (PPA** **spillage)**: H_2_ is produced only when the campus electricity consumption is fully met with wind electricity (eq 14), leveraging excess electricity from the PPA. The electrolyzer operation is capped by the electrolyzer size, *S*_E_ [in (MW_IN_)].
14 $$E_{\mathrm{El}}=\min\left(S_{E},\max\left(E_{\mathrm{PPA}}-D_{E}\right),0)\right)$$
  We also vary the wind PPA limit as a parameter. As we increase the limit, the electrolyzer is more frequently used. This alternative can inform future scenarios in which selling electricity to the grid is possible.
- **Alternative 3 (Hi-SOEC with CCUS):** Similar to Alternative 1, but with the use of CCUS to mitigate natural gas combustion emissions.
- **Alternative 4 (PPA spillages and CCUS):** Similar to Alternative 2, but with the use of CCUS.
- **Alternative 5 (purchasing H**_***2***_ **):** Rather than developing an on-site SOEC, the DES will purchase H_2_ fuel.

#### Parametric Analysis for the Levelized Cost of Multiple Products

To explore alternatives, we used parametric analysis to study design choices and product prices. We examined the financial performance of several design alternatives by calculating the levelized cost of each product, without relying on traditional allocation approaches or assumptions on the value of our diverse outputs. The design alternatives included different electrolyzer sizes, *S*_E_ [in (MW_IN_)], percent of H_2_ that the CHP can use as fuel, denoted *p* [in (%)], and modifications to *E*_PPA,Max_. We also performed parametric analyses for the levelized cost of the four products by calculating the levelized cost of one product, keeping the prices of the other three fixed. By systematically changing these fixed parameters, we repeat levelized cost calculations to explore and define breakeven curves that map out regions of economic viability. We selected literature-based ranges for commodity prices of $0.02–5.5/_kgO2_,^53^ $1–10/_kgH2_,^20,24,28,74,75^ $0–130/_TonneCO2‑red_,^57,76–78^ $0–80/_TonneCO2‑use_.^68^ In our default case, we evaluate a SOEC of 3.4 MW_NET‑INPUT_ (or 100 kg/h), the CHP fuel blend accepting up to 30% H_2_, and a 50 MW PPA operating under Hi-SOEC with CCUS.

## Results

### The 8760 Model: Annual Operation

The central plant supplied 76% of the electricity load and 70% of the heat demand of the DES in our analysis. During our study period, the DES consumed 512 GWh of electricity and 1.38 million MMBTU of thermal demand (leading to peaks load of 97 MW and 365 MMBTU/h). The plant supplied 385 GWh and almost one million MMBTU. The rest of the electricity demand was supplied by the PPA (120 GWh, 23%) and grid (6.5 GWh, 2%). The electricity spillage was 441 MWh (or 3.70% of the annual PPA supply) and the boiler generated 412,000 MMBTU/yr, covering 30% of heat needs.

The CHP operates close to average efficiencies reported for similar facilities. However, given the large variations in heat and electricity demand over the year, the CHP operates across a wide range of operating points and efficiencies. We show the energy demand for the DES and wind PPA production throughout the year in SI Section 7 and Figure S4.

### Technoeconomic Evaluation

#### Consequences of SOEC Deployment

Both the size of the electrolyzer and the decision to deploy CCUS are positively correlated with the system’s electricity consumption. Electrolyzers between 1 and 10 MW_NET Input_ (∼30–300 kg/h) operating at a capacity factor of 0.85, increase annual electricity demand by 1–10% (up to 60 GWh). CCUS deployment increases electricity consumption from the grid by 125 GWh the grid electricity consumption and allows capturing of 250 kTonne_CO2_.

The base-design project with Hi-SOEC is close to the breakeven point with additional net annualized costs of $0.06 M (Figure 2A). The CCUS is the most significant cost ($7.8M/yr), followed by increased gid electricity costs ($5.4M/yr), and CCUS transportation and storage costs ($4.9M). Natural gas, SOEC annualized cost for a 3.4 MW-SOEC, and SOEC Stack replacements are relatively small annual CAPEX costs ($1.1M, $0.8M, and $0.6M, respectively). The existence of a carbon market or pricing is necessary to meaningfully defray these costs with annual revenues of $12.5 M to capture and sell carbon, $8.5 M in avoided carbon taxes, and $1.4 M in averted O_2_ purchases.

**Figure 2:**
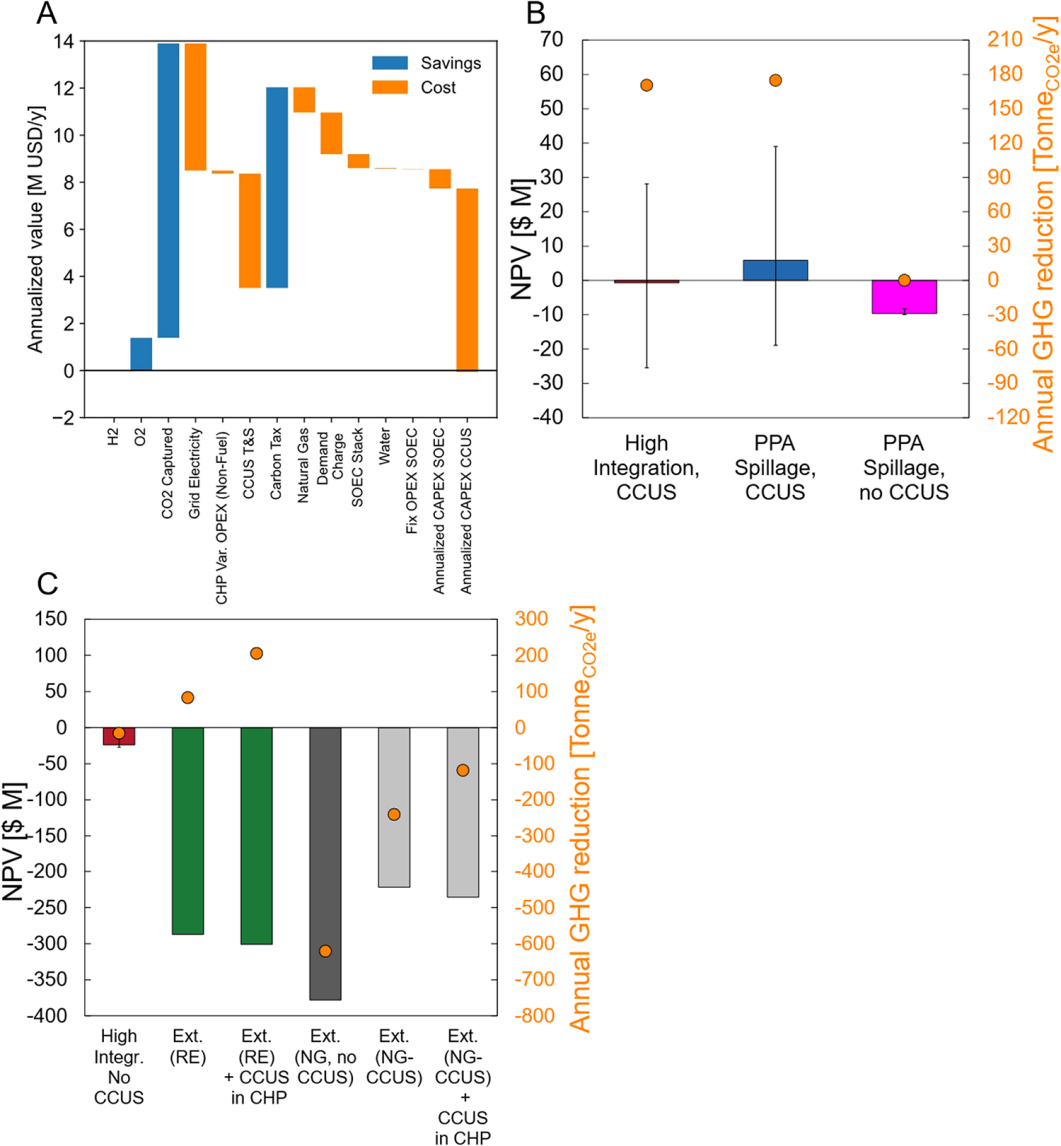
Base-design projects prescreening. (A) Annual worth of Hi-SOEC with CCUS under default commodity prices. (B) NPV of base design under High Integration with CCUS and PPA Spillage alternatives, and (C) High Integration without CCUS and Purchasing alternatives. For panels (B, C), bars correspond to NPV (left axes), and orange circles correspond to annual CO_2e_ emission abatement (right axes). Error bars show extreme PPA and demand scenarios for alternatives with H_2_ production. Reproduced and modified with permissions from ref 59. Copyright 2025 Diego Hincapie-Ossa.

#### Screening Business Model Alternatives

The base design exhibits large variability in financial performance under different business model alternatives (Figure 2B). Alternative 4 (PPA spillage with CCUS) is the only net positive alternative (NPV of $ 5.83M) with GHG reductions (175 kTonne/yr), with respect to the DES operation before the project, including direct emissions and indirect emissions for electricity production. Alternative 3 (Hi-SOEC with CCUS) has a negative NPV (-$0.70M) but significant emission reductions (170 kTonne/yr), making it more preferred than Alternative 2 (PPA spillage without CCUS) with an NPV of -$9.6 M and emissions reductions of 0.02 kTonne/yr. Although these two alternatives are net financial losses, their performance is more sensitive to variation on product prices than Alternative 5 (Purchasing H_2_). Alternative 1 (Hi-SOEC integration without carbon capture) incurred losses (-$24M) and led to higher CO_2_ emissions (15.2 kTonne/yr). In this group, only externally buying green H_2_ substantially reduced emissions (green bars in Figure 2C).

Alternatives have different product mixes, driving their sensitivities. Hi-SOEC have significant O_2_ revenues while no alternatives have substantial gains from hydrogen sales (itemized profits and losses listed in the SI Section 8, Table S4). Implementing CCUS in the central plant reduces emissions when H_2_ production occurs on-site. However, CCUS deployment fails to reduce the carbon footprint of externally supplied natural-gas based H_2_ (gray bars in Figure 2C). We also find that CCUS exacerbates projects’ sensitivity to wind and electricity demand, as shown by the larger error bars for projects with CCUS and on-site H_2_ generation (Figure 2B,2C).

#### Parametric Levelized Cost Analysis

Figure 3 shows the required carbon price (i.e., levelized cost of carbon abatement) over O_2_ price for two on-site H_2_ supply alternatives. Parallel lines correspond to alternatives at different prices for captured carbon (note that captured carbon refers to material CO_2_ captured by the CCUS and sold which is not revenue from avoiding emissions). CCUS-enabled designs are sensitive to variations in the price of captured carbon. PPA spillage projects with CCUS (continuous blue lines in Figure 3A) are less sensitive to O_2_ prices for the base design (evidenced by the less pronounced slope). Conversely, Hi-SOEC projects (red lines in Figure 3A) are more sensitive, with viability occurring at >$3.2/kg_O2_ (and captured carbon has zero price).

**Figure 3:**
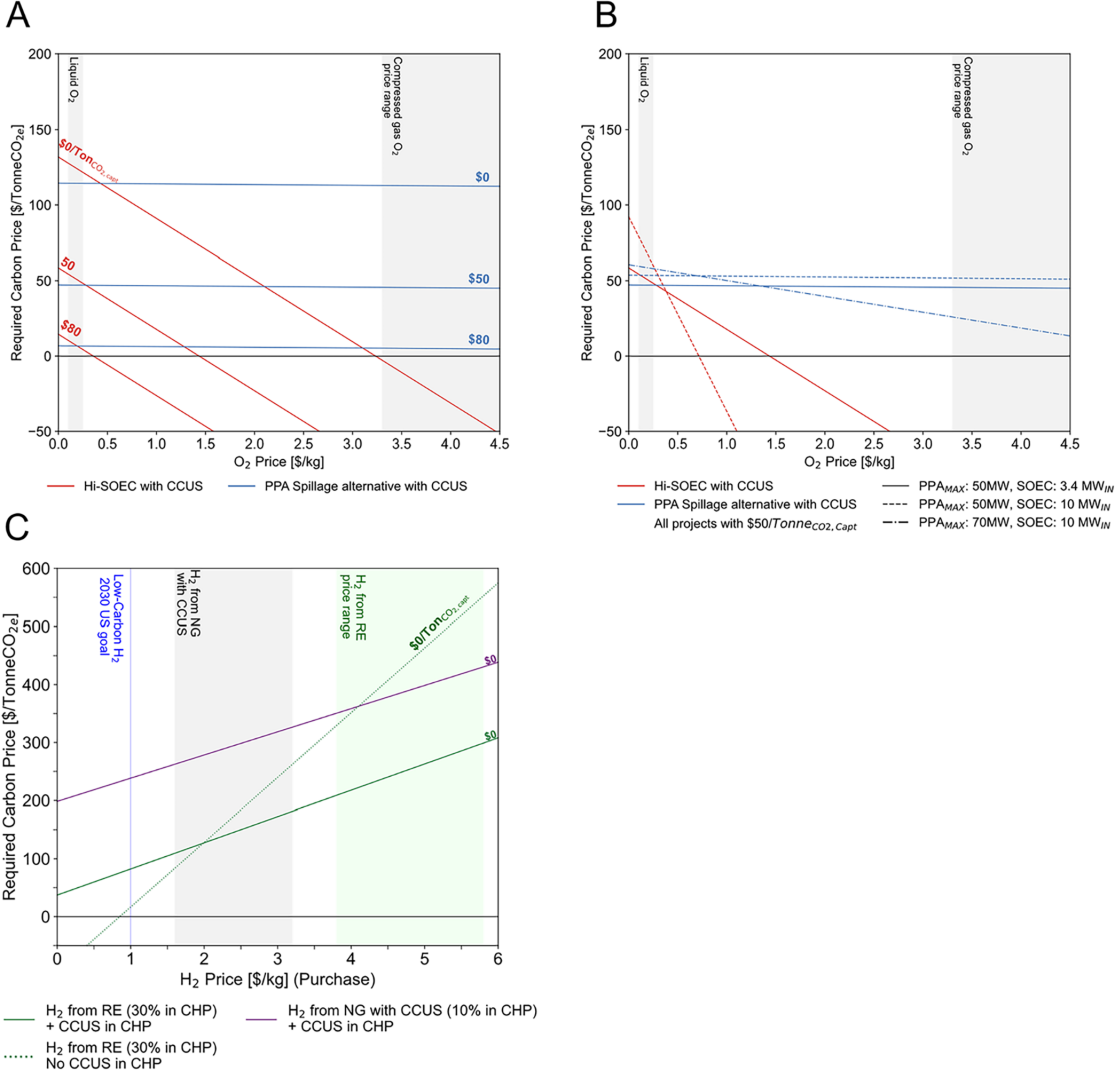
Required carbon price as functions of O_2_ price variations for base design. (A) PPA Spillage with CCUS and Hi-SOEC. (B) Variations of size and PPA. (C) Sensitivities of Purchasing H_2_ alternatives to H_2_ price. Shaded regions represent current market conditions. Reproduced and modified with permissions from ref 59. Copyright 2025 Diego Hincapie-Ossa.

Design variations dramatically affect certain project viability profiles. Increasing electrolyzer size to 10-MW_in_ makes the evaluated Hi-SOEC project more sensitive to O_2_ prices (red dotted line in Figure 3B). So long as oxygen prices are greater than $0.72/kg_O2_, we do not require carbon prices. In contrast, PPA spillage does not increase sensitivity to O_2_ prices with a larger electrolyzer (as the slope is less steep) unless a larger PPA is implemented (dash-dotted blue line in Figure 3B).

H_2_ prices are critical in the viability of purchasing alternatives. We assessed two green H_2_ purchasing projects (Figure 2C) and purchasing “blue” H_2_ to be 10% of the central plant’s fuel mix. We selected the latter instead of the base design (fuel mix of 30% H_2_) which increased carbon emissions. Despite lowering carbon emissions, these three projects were net economic losses for the base-design. However, we observe dissimilar sensitivity to H_2_ prices with purchasing green H_2_ to serve a DES that does not implement CCUS (dashed green line markers in Figure 3C) having the steepest slope of these three alternatives. Without carbon-capture related prices, if H_2_ is priced at $1/kg (“Earth Shot” US goal by 2030),^74^ carbon prices must be >$16/Tonne_CO2e_ to be viable.

Annual cost and revenue structures vary substantially across alternatives. Alternatives 3 and 4 benefit from carbon prices (e.g., Alternative 4 had revenues of $11.8 M from captured carbon and $8.74 M for reduced emissions) while Alternative 1 benefited from oxygen sales ($1.39M)–even at competitive liquid oxygen prices ($0.2/kg) and higher natural gas purchase costs ($1.1 M). The purchasing H_2_ alternatives reduced natural gas costs by more than $7M/yr, although this is offset by the costs H_2_ fuel ($9.8–37.1M). This is consistent with past work that found that natural gas represents 1/2 to 1/6 of the cost of gas-derived H_2_ and H_2_ from electrolysis ($14–35 ^20^ vs $6/MMBTU).

## Discussion

### Higher Resolutions and Complexities Improves TEA Quality

The use of highly-resolved models allowed us to identify system-level behavior that TEAs parametrized with annual averages cannot. Since SOEC or CCUS projects substantially modify DES operation, understanding and identifying second-order, system-level effects is critical given nonlinearities that cannot be measured with seasonal or annual resolutions. For instance, we can measure the benefits of increasing the PPA capacity limit in Alternative 2 (PPA spillage), as our model can observe the frequency and magnitude of spillage. We determined that a 40% increase in the PPA (to 70 MW) increases excess electricity in the base year by a factor of 25 (>10 GWh vs 0.4 GWh).

Fundamentally, there is substantial information gained from increased resolution. Our detailed model had substantial differences with the results of a side analysis using constant central plant thermal and electrical efficiencies (reported in SI Section 9 and Table S5). Using fixed efficiencies overestimated natural gas consumption for the Hi-SOEC base-design project. We obtained a 21.6% lower fuel consumption compared to the best-case benchmark of simultaneously using the maximum thermal and maximum electrical efficiency. This counterintuitive result demonstrates that MFSs can have higher efficiencies as simultaneous production of electrical and thermal energy is more efficient that their production in dedicated, separate processes. This fuel use discrepancy leads to higher estimated costs of $312,000/yr compared to our model, about 50% of the annual losses in the Hi-SOEC base design.

Critically, our results challenge a common assumption in TEAs of constant average efficiencies.^38^ Our results show how leveraging efficiency curves to create models that calculate efficiency at hourly resolutions can better assess variable operational conditions. Given increased data on industrial processes, data with finer temporal resolution will support these types of assessments. This practice can substantially improve TEAs’ accuracy–and ultimate usability.

Furthermore, increasing model complexity to analyze system-level effects is essential in demonstrating viability. In the analyzed DES, second-order effects govern physical flows that determine system performance. For instance, we determined that the base-design project under the Hi-SOEC without CCUS alternative is not economically viable as the DES combusts H_2_ produced with energy from the DES itself, leading to H_2_ round-trip inefficiencies.^79^ Similarly, we found that purchasing blue H_2_ to replace 30% of the central plant’s fuel needs increased DES emissions, performing substantially worse than only combusting a 10% H_2_ mix. In this case, displacing a larger amount from gas does not offset life-cycle environmental impacts of the H_2_, mainly generated in the fossil-derived hydrogen production (carbon intensity of 4.6 kg_CO2_/kg_H2_ ^24^) and the required energy to run the CCUS.

### Multifunctionality is Pivotal to Determine Economic Viability

The benefits from H_2_-based MFSs lie in the interactions between products. As product mixes vary across alternatives, studying the levelized cost of a single product is insufficient to determine economic viability. We find that the Hi-SOEC alternative can benefit from high O_2_ prices. As can be seen in Figure 2A and the analysis in SI Section 8, the viability of projects with carbon capture depended on carbon prices. Alternatives with the highest on-site H_2_ production from electrolysis coproduce O_2_, which creates meaningful revenues in markets with high oxygen prices. For example, in a scenario without carbon price, Alternative 3 (Hi-SOEC with CCUS) is viable at $3.2–4.5/kg_O2_, a realistic range for hospitals with a small number of beds sourcing oxygen gas.^53^ In our case study, since the DES hospital purchases liquid O_2_, the same project design can be viable with a $50/Tonne carbon abatement tax combined with carbon capture tax credits worth $60/Tonne.^80^ In SI Section 10, we provide an example of a (linear) mathematical representation for possible combinations of two product prices making the alternative viable.

Although H_2_ prices do not influence the economic performance of on-site production alternatives (that can be viable with other products’ revenues), H_2_ prices are a key determinant when purchasing H_2_, especially in combination with carbon prices. The three projects shown in Figure 3C require high (>$209/Tonne_CO2_) carbon-related revenues to become viable with current hydrogen prices (shaded areas). Furthermore, in carbon capture cases, CCUS CAPEX and electricity-consumption OPEX offset the benefits of purchasing H_2_ at low prices, leading to negative NPVs. Critically, alternatives that incorporate the CCUS invariably depend on related prices or revenues–even in an extreme case of free H_2_. In contrast, projects with green H_2_ purchases and no CCUS at the central plant (dotted line in Figure 3C) are more viable at lower H_2_ prices, breaking even at $0.85/kg_H2_ in scenarios without carbon price.

### Policy Elements Dominate the Influence of Technological Factors

Besides the influence of product prices and sensitivity to energy demand and wind availability (Figure 4A and discussed in SI Section 11), policy and market conditions substantially influence project viability. While research will continue to improve relevant technologies, system-wide assessments are necessary complements as nontechnological parameters can have larger effects. From our sensitivity analysis of project design parameters (SI Section 12), we find that policy incentives (i.e., carbon prices) are more critical than technology cost and performance variables.

**Figure 4:**
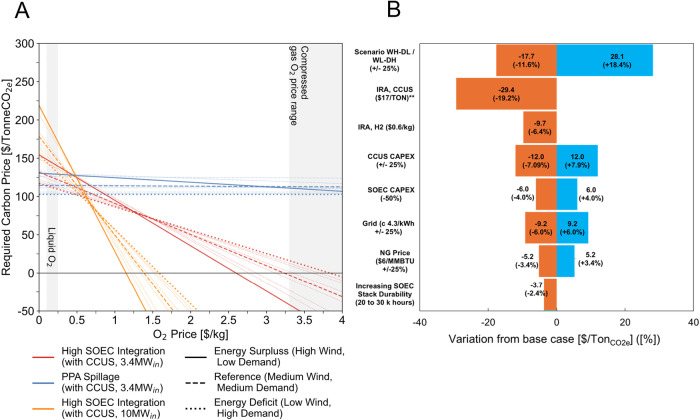
Uncertainty analysis across O_2_ prices. (A) Uncertainty profile for the minimum carbon price as a function of the oxygen price. Alternatives present specific uncertainty profiles which vary depending on the market where they operate, where steeper slopes indicate more sensitivity. (B) Sensitivity Analysis for Alternative 3 (Hi-SOEC with CCUS), at an oxygen price for large hospitals like that in the DES ($0.2/kg_O2_).^53^ Reproduced and modified with permissions from ref 59. Copyright 2025 Diego Hincapie-Ossa.

Policy mechanisms that address pull factors may be more impactful than addressing the push factors of technical performance. Incentives for carbon management can be critical to guarantee economic viability with a moderate tax credit ($17/Tonne_CO2,capt_) reducing minimum carbon prices by 16.1% (Figure 4B) (although larger tax credits^81^ may be necessary for some projects). Conversely, efforts to reduce SOEC capital costs and cell stack durability are relatively less important, as their impacts on project viability are marginal (−3.4 and −2.4% in the studied cases).

## Conclusions

Traditional analytical tools, like TEA, are commonly used for single-product systems – but, as we do here, TEAs informed by temporally resolved data, can examine multiple products and consider effects of their prices in combination, by calculating levelized costs of products as functions of other products’ costs. Our approach allowed us to assess the value of emerging technologies like SOEC and products like H_2_ that can be used in a variety of ways. Products that are not currently competitive for standalone production can become competitive in MFSs by leveraging economies of scale. Furthermore, many of these systems are close to the breakeven point, and neglecting second-order effects not captured at coarse resolutions may lead to premature decisions to abandon MFS projects. Our work contributes to formalizing a method for quantifying those cobenefits.

In this work, we identified product price ranges for H_2_ that make SOEC projects economically viable, considering coproduct revenues. Critically, coproduced oxygen can make SOEC projects that generate large amounts of hydrogen viable, while revenues from carbon reduction are needed to make CCUS viable. Hydrogen prices do not influence cases in which the system produces H_2_ for itself but are critical when purchasing H_2_. Purchasing H_2_ from renewable electricity-based electrolysis can become viable without carbon prices with H_2_ prices ∼$1/kg–except when CCUS is implemented in the DES. Even if low-carbon H_2_ prices fall, as is expected in the coming decades, DESs that implement CCUS will still require carbon prices to be viable. Policy mechanisms that support low-carbon H_2_ and CCUS are also critical for these systems.

Our approach leverages available industrial data but can be improved with more detailed process modeling (e.g., using historical CHP operational fixed costs) or data for multiple years to analyze a wider range of operational conditions. The further use of high-resolution data for other processes (e.g., boilers) can also improve model accuracy, and optimization approaches can better inform the technical potential of the projects. Finally, our model can be improved as real operational data become available for the SOEC and CCUS technologies.

Taken together, our work emphasizes that system-level perspectives are critical to accurately assess technoeconomic and environmental performance of emerging products, especially in MFSs. Moreover, the use of increasingly available data on real information at temporal resolutions relevant to stakeholders can be crucial to determine if a project is economically viable.

## Supplementary Material



## Notes

Selected TEA literature and trends for H_2_ MFS literature; main equipment description in the District Energy System; capacity and transmission charges of the DES; analyzing the benefits of higher-temporal resolution data: Combined Heat and Power (CHP) efficiency curve model and operation profile; technoeconomic parameters of cost model for different alternatives; electrolyzer escalation factors; operation profile of the District Energy System; analyzing alternatives for the base-design project; results of model complexity analysis; determining the relationship for the levelized costs of O_2_ and carbon prices in a specific project; examining the Uncertainty to Wind and Energy Demand; scenarios for market and operational conditions analyzed in sensitivity analysis (PDF)

## #

Oak Ridge National Laboratory, Oak Ridge, Tennessee 37830, United States

## Symbols

H_2_: hydrogen
O_2_: oxygen
*C*_E_: cost of electrical services [$]
*S*: electrolyzer size [MW_IN_]
*C*_Trans_: transmission charges [$]
*R*: revenues
Tonne: metric tonnes
*E*: electricity supplied by the PPA [MWh]
*F*: fuel
*Q*: heat [MMBTU]
*K*: cost coefficient [$/unit]
*P*_Trans_: peak transmission [MW]
*P*_Cap_: representative annual peak transmission [MW]
*D*: demand
*C*_f_: capacity factor (% of installed capacity units)
η: efficiency
Δ*H*: total energy requirements in electrolyzer
Δ*S*: heat requirement of electrolyzer
Δ*G*: Gibbs energy, electrical requirement of electrolyzer
WH-WM-WL: wind high, wind medium, wind low (scenarios)
DH-DM-DL: demand high, demand medium, demand low (scenarios)

## Subscripts:

CHP: metric or process associated with the combined heat and power plant
CCUS: carbon capture, utilization, and storage
NG: natural gas
B: boiler
Th: thermal
El: electrical
*t*: superscript associated with period “*t*” in a specific hour
Base: baseline
Trans: associated to transmission charges
Cap: associated to capacity charges
Var: variable cost
SM: escalation factor of a component
W: wind
Hr: high resolution (hourly)
Avg: annual average
BaU: business as usual
