# Supplementary material for: Benefits of Temporally-Resolved, Policy-Relevant, Data-Informed Technoeconomic Evaluation of Multifunctional Systems: H2 Deployment in a District Energy System
Source: ACS Sustain Chem Eng. 2026 Apr 23;14(17):8001–11. doi: 10.1021/acssuschemeng.5c07425 (PMC13148720; doi:10.1021/acssuschemeng.5c07425)
Supplement: Supplementary file 1 [file sc5c07425_si_001.pdf]

Supporting Information

for

Benefits of Temporally-Resolved, Policy-Relevant, Data-Informed Technoeconomic Evaluation of Multifunctional Systems: H2 Deployment in a District Energy System

Diego A. Hincapie-Ossa,[1,°] Chris Swanson[2], Daniel B. Gingerich[1,3,4,5,*]

[1] Department of Civil, Environmental and Geodetic Engineering, The Ohio State University, Columbus, OH, 43210
[2] Engie North America, Columbus, OH, 43210
[3] Department of Integrated Systems Engineering, The Ohio State University, Columbus, OH, 43210
[4] Environmental Science Graduate Program, The Ohio State University, Columbus, OH, 43210
[5] The Sustainability Institute, The Ohio State University, Columbus, OH, 43210

[°] Author's Current Affiliation: Oak Ridge National Laboratory, Oak Ridge, TN, 37830

* Author to whom correspondence should be addressed. E-mail: gingerich.62@osu.edu, Phone Number: (614) 688-1768

This supporting information contains 22 pages (including references), five tables, and four figures. It comprises the following sections: (1) Selected TEA literature and trends for H_2 MFS literature; (2) Main equipment description in the District Energy System; (3) Capacity and Transmission Charges of the DES; (4) Analyzing the benefits of higher-temporal resolution data: Combined Heat and Power (CHP) efficiency curve model and operation profile; (5) Technoeconomic parameters of cost model for different alternatives; (6) Electrolyzer escalation factors; (7) Operation profile of the District Energy System; (8) Analyzing alternatives for the base-design project; (9) Results of model complexity analysis; (10) Determining the relationship for the levelized costs of O_2 and carbon prices in a specific project; (11) Examining the Uncertainty to Wind and Energy Demand; (12) Scenarios for market and operational conditions analyzed in sensitivity analysis.

1. Selected TEA literature on H_2-based MFS and publication trends

From our literature review, we found an increasing trend of works analyzing the technoeconomic viability of multifunction H_2 and diverse approaches to deal with price interactions of multiple products. With the following prompt in Google Scholar, we identified 44 papers from 1994 to November 2024:

> allintitle: ("Hydrogen" OR "H2") AND ("Multifunction" OR "Multi-function" OR "Multi-functional" OR "Multifunctional" OR "Polygeneration" OR "Co-production" OR "Coproduction" OR "Integrated") AND ("Technoeconomic assessment" OR "TEA" OR "Techno-economic Assessment" OR "Techno Economic Assessment" OR "Levelized" OR "Levelised" OR "Cost")

In Figure S1, we report publication trends on the topic showing a steepen increase in the past five years. In Table S1, we include descriptions of the analysis product-price interactions and uncertainty modeling.

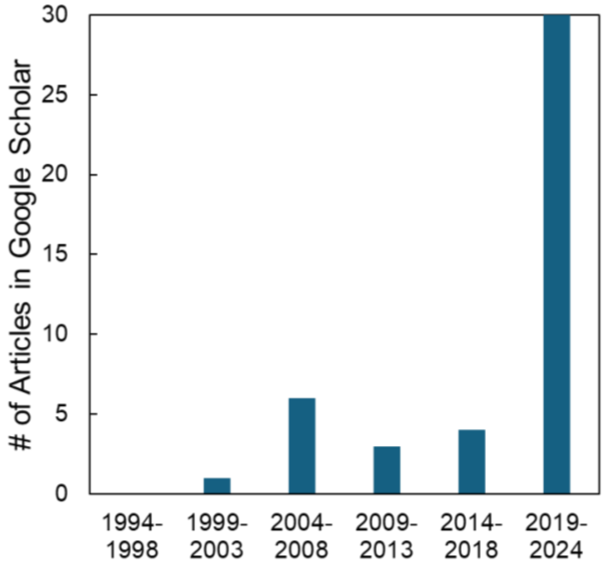

Figure S1. Literature trends on H_2-based MFS. There is a steepen increase in the Technoeconomic Assessment literature analyzing these systems.

Table S1. Selected recent literature on H_2 – based MFSs with description on choices about multi-product assessment and uncertainty analysis.

Year	Work	Metric	Primary Product / Service		Secondary Products / Service		Interaction between costs	Uncontrollable variables and empirical parameters values		System Design		Sensitivity analysis	
			Cost/ Price handling	Product	Cost/ Price handling	Product							
	This Work	NPV, Levelized Cost (LC)	LC (parametric on cost of other products services)	CO_2 Reduc	LC (parametric on cost of other products services)	O_2, CO_2Capture, H_2	Parametric in LCO-O_2, -Carbon Reduction, -Carbon Capture and -H_2	Scenarios	Demand, Wind year profile	Parametric	Size, PPA, H_2 accepted in Turbine	One at a time	Demand, wind profile, IRA, grid elec. Cost, natural gas cost
2024	Sochanski et al.[1]	IRR	Not reported / Fixed parameters	Electricity	Fixed	H_2, Hot water	Scenarios	Not reported / Fixed parameters		Not reported / Fixed parameters		Scena-rios	Carbon costs ETS (EU)
2024	Safder et al.[2]	Cost	Not reported / Fixed parameters	Methanol	Fixed	H_2, Power, Cooling water	Fixed	Not reported / Fixed parameters		Not reported / Fixed parameters		NA	
2024	Acen et al.[3]	LC	LC	Electricity	LC	H_2, Heat	Joint calculation	Not reported / Fixed parameters		Parametric (Optimization)		Para-metric	
2024	Wang et al.[4]	LC	LC (parametric on cost of other products services)	H2	Input parameter	Electricity	Parametric analysis H_2 vs Electricity product	Parametric	Technical parametres, escalation factor, DR, lifetime	PAR	Size, technical parameters	NA	
2023	Giwa et al.[5]	IRR	Not reported / Fixed parameters	Ethanol, H2, Bio-Oil	Fixed		Fixed	Probabilistic (Montecarlo)		Included in probabilistic	Size	One at a time	Costs, price of Ethanol
2021	Ng et al.[6,7]	LC	LC	H2	Fixed	Waste recovery, Electricity	Fixed	Not reported / Fixed parameters		Not reported / Fixed parameters		NA	
2021	Cao et al.[8]	LC	LC	Electricity	Not reported		Only one product analyzed						

2019	Jiang et al.[9]	LC	LC	Electricity	LCOH (Joint calculation)	H2	Joint calculation			Scenarios	Different technologies	Parametric	Tech pars
2019	Szima and Cormos[10]	LC	LC	Electricity	LC	H2	Scenarios	Not reported / Fixed parameters		Scenarios	3 cases	One at a time	Costs, DR, Availability
2019	Spallina et al.[11]		LC	H_2	Not reported / Fixed parameters	Electricity, CO_2 Reduction	Scenarios in Sensitivity Analysis	Not reported / Fixed parameters		Not reported / Fixed parameters		Para+metric	Varying cost
2015	Cormos[12]	LC	LC	Electricity	Not reported / Fixed parameters	H_2, CO_2 Reduct. / Capture	Fixed	Not reported / Fixed parameters		Scenarios	4 cases different technologies	One at a time	Costs, DR, Availability

2. *DES main energy equipment description*

A Combined Heat and Power plant (CHP) and the system of boilers are the main sets of equipment producing electricity and heat in the DES. These systems are complemented by external sources following priority operation rules shown in Figure S2.

Boilers: A set of industrial boilers contribute additional 400,000 lb/h of steam with a delta enthalpy of 1123 BTU/lb.

CHP: The CHP plant is a combined cycle with two combustion turbines and one steam turbine adding up to 100.4MW nameplate capacity. The combustion turbines operate with natural gas and can operate with 15% of H2 in the fuel mix without retrofit (and up to 30% of H_2 with retrofits). The CHP can produce up to 300,000 lb/h of steam with flexibility to vary the load using the Heat Recovery Steam Generator (HRSG) duct fire.

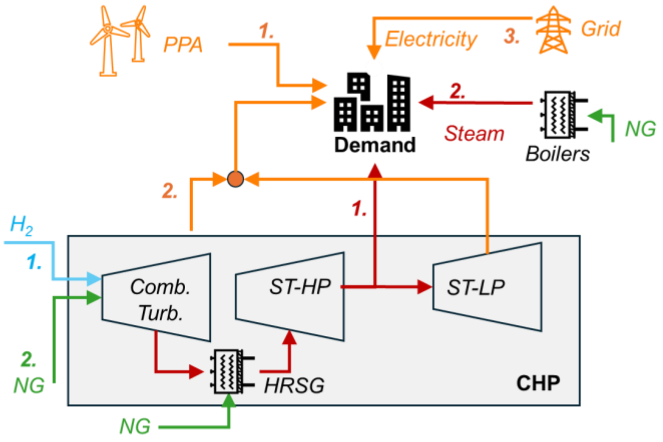

Figure S2. Schematic of DES energy-supply operation rules. The system has redundant supply for electricity and heat. After implementing a H_2 integration project, the CHP has two fuel options to operate. The numbers in the schematic show the order of entrance in operation; however, there are operational limits that must be fulfilled (e.g., limits of the PPA, equipment capacity limits and operational ranges of the CHP.

3. Capacity and Transmission Charges of the DES.

The transmission costs (Eq. *S1*) are calculated as observed monthly peak load, P_{TRANS} (in [MW]), multiplied by the corresponding monthly transmission rate K_{TRANS} (in [$/MW-mo]).

$$C_{trans} = \sum_{n=1}^{12} P_{Trans}^{Mo} K_{Trans}^{Mo} \qquad Eq.\ S1$$

Meanwhile, given the impossibility of predicting demand at regional peaks, we estimate capacity cost (Eq. *S2*) as the annual peak load, P_{Cap} (in [MW]), in August – the month with the five peak hours for the DES in our case-study year – multiplied by the average monthly zonal capacity price, K_{Cap} ([in/MW-day]), in 2022 and number of days per year.

$$C_{Cap} = 365 * P_{Cap} K_{Cap} \qquad Eq.\ S2$$

4. Analyzing the benefits of higher-temporal resolution data: CHP efficiency curve model and operation profile

The energy demand profile and the DES operational rules require the CHP to operate in different efficiency levels. We developed an efficiency curve using information of electric and thermal load and fuel consumption of the plant to calculate its combined thermal and electric efficiency for each operation point (schematically represented in Figure S3). With this two-dimensional curve we calculate the fuel consumption of the CHP with hourly resolution. The overall efficiency in our model – calculated using a turbomachinery modeling and design software – differs from the weighted average of the thermal and electric efficiencies. Given the wide range of operation points required during the year for the CHP to cover the varying heat and electricity demand (illustrated in Figure S4A) we expect a large variance on the efficiency values along the year.

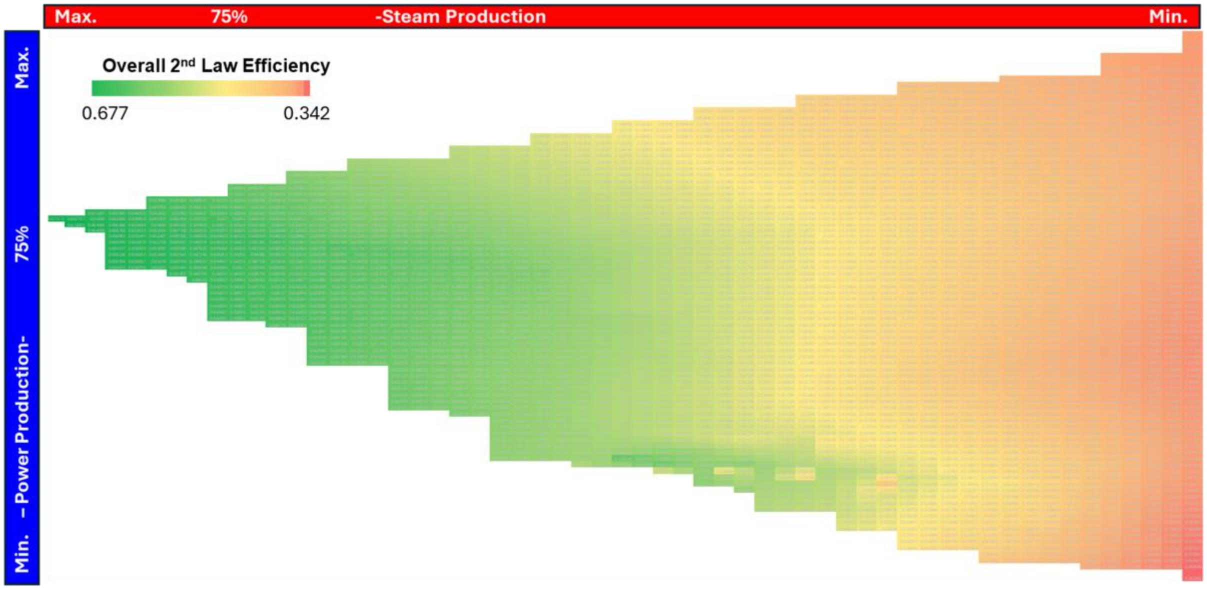

Figure S3. Map of efficiencies for different operation points of the CHP.

We quantify the CHP fuel consumption, $F_{CHP_{Hi}}$ (in MMBTU/y), using efficiency values for each hour h, η_{CHP_h} (in percent) and hourly heat consumption, Q_{CHP_h}(in [MMBTU/h]) and electricity generation, E_{CHP_h} (in [MW]), corresponding to specific DES conditions of demand and supply for each hourly period. Finally, we compare the results of our model with those from a fixed-efficiency calculation (Eq. *S3*), in which we use a design or representative CHP efficiency η^*_{CHP} and annual thermal energy production, Q^*_{CHP} (in [MMBTU/y]), and electricity E^*_{CHP} (in [MWh/y]).

$$\Delta F = F_{CHP_{Hi}} - \hat{F}_{CHP} = \sum_{h=1}^{8760} \frac{(Q_{CHP_h} + 3.412\, E_{CHP_h})}{\eta_{CHP_h}} - \frac{\hat{Q}_{CHP} + 3.412 \hat{E}_{CHP}}{\hat{\eta}_{CHP}} \qquad Eq.\ S3$$

5. *Technoeconomic parameters of cost model for different alternatives*

We assess the *base design* under five alternatives and then ran parametric analyses to assess the combined levelized cost of its products. We present in Table S1 a list of parameters we considered for these analyses, and for uncertainty and sensitivity analysis.

Table S2. project parameters in base case, in parametric levelized cost calculations, and in sensitivity analysis.

Parameter	Baseline (No H_2 Project)	Base Design and Nominal Conditions	Parametric Analysis	Sensitivity Analysis
CHP power gen. capacity ($E_{CHP,Max}$)	100.4 MW_{Net}	100.4 MW_{Net}	100.4 MW_{Net}	
Project Life	20 years			
Analysis Year	2024			
CHP steam gen. capacity ($Q_{CHP,Max}$)	Depends on CHP electrical load			
CHP Non-Fuel Variable costs	$\$2.9/MWh$			
Electrolyzer capacity (S_E)	0	3.4 MW_{IN}	1-10 MW_{IN}	10 MW_{IN}
H_2 percent in CHP fuel mix (p)	0	30%	10 - 30%	
PPA limit (PPA_{Max})	50 MW	50MW	50-70 MW	
SOEC CAPEX	N/A	$\$2541/kW_{IN}$ (reference: 5-MW_{IN} electrolyzer)		$\$1273/kW_{IN}$
SOEC Stack durability	20,000 h			30,000 h
SOEC Stack cost	$\$ 400,000 / MW$			
SOEC Var OPEX ($C_{SOEC,VAR}$)	3% of CAPEX			
CCUS CAPEX	N/A	$\$89.3$ M**		+/-25%
CCUS Operational parameters	N/A	Capture Rate: 85%<br>Energy Penalty: 25% of power output<br>Transportation and storage costs: $\$19.43/Tonne_{CO2}$		
Carbon intensities puerchased H_2:	N/A	H_2 from Natural gas without CCUS: 10 kg_{CO2}/kg_{H2}<br>H_2 from Natural gas with CCUS: 4.6kg_{CO2}/kg_{H2}		
Discount Rate	N/A	6%/y		

Alternatives for SOEC operation		Five alternatives:<br>• High Integration with CCUS<br>• High Integration w/o CCUS<br>• PPA Spillage with CCUS<br>• PPA Spillage w/o CCUS<br>• External H_2 purchase	Pre-screened alternatives:<br>• High Integration with CCUS<br>• PPA Spillage<br>• PPA Spillage with CCUS	
NG price (K_{NG})*	$6/MMBTU			+/- 25%
Elec. Price* (E_{Grid})	$43/MWh			+/- 25%
Water use (withdrawal)* (c_{H2O})	$3.72/Tonne			
Monthly Zonal Capacity price ($Kcap$)	$142.16 / MW for the months in the first semester of the year and $50.05 / MW for the second semester.			
Monthly transmission rate (K_{Trans})	$6.72 MW-mo for the first semester and $7.83 / MW-mo for the second.			
H_2 buying price for external H_2 supply alternatives	N/A	• Coal w/o CCUS: $ 2.58/kg<br>• Coal, CCUS: $ 3.09/kg<br>• NG w/o CCUS: $ 1.06/kg<br>• NG with CCUS: $ 1.64/kg<br>• RE electrolyzer. (low) $4/kg<br>• RE electrolyzer. (high) $8/kg	N/A	N/A
IRA[13]	No IRA	No IRA	No IRA	H_2 / CCUS
Wind profile	Reference	Reference	Reference	Ref. +/- 25%
Heat demand	Reference	Reference	Reference	Ref. +/- 25%
Electricity demand	Reference	Reference	Reference	Ref. +/- 25%
LCOH<br>LCO-O_2<br>LCO-CO_2 avoided (Carbon price)<br>LCO-CO_2 captured	N/A	N/A. Financial performance calculated as the NPV.	LCs are calculated using the prices of the other three products as inputs (e.g., for the LCOH, we use the prices of O_2, CO_2 avoided, and CO_2 captured as inputs)	
H_2 selling price	N/A	$ 1 / kg	*[1-10 $/kg]*[14–18]	
O_2 price		$ 0.2 / kg	*[0.02-5.5 $/kg]*[19]	

Carbon price		$ 50 / kg	*[0-130 $/Tonne]*[1,11,20,21]
CO_2 selling price		$ 50 / kg	*[0-130 $/Tonne]*[22]

* Utility prices for the DES reported by the operator
**Calculated as 76%[23] of a 100-MW natural gas combined power plant using a reference price of $1.176/kW.[24]

6. Electrolyzer Escalation Factors

We estimated escalated costs based on variations of SOEC sizes. We considered a base capital cost of \$2,541/kW_{IN} in 5-MW electrolyzers and escalation factors for the main SOEC components as in Table S3.[25]

Table S3. Escalation Factors of key SOEC components and calculated factor for the system.

Capacity Scalation (Economies of Scale)	Weight in Cost and in Escalation Formula	Escalation Factor	Reference
Stack	30%	0.87	Böhm et al. (2020) [25]
BoP	34%	0.73	
Power Electronics	30%	0.75	
Gas Conditioning	6%	0.6	

7. Operation Profile of the District Energy System

The CHP operates close to efficiencies reported for similar co-generation plants throughout the entire year, as operating guidelines are designed to keep the system operating in areas of acceptable efficiencies. The plant operated fifty percent (i.e., the 25th and 75th percentile) of the time between efficiencies of 0.52 and 0.59 (5th-95th percentiles of 0.51-0.61). Seven months had average efficiencies above the annual median hourly efficiency (0.549) with the winter months having the lowest efficiencies. In those months, the plant had 816 unavailable hours, as the required heat and electricity was outside efficient operation levels. As shown in Figure S4B, the operation conditions took the plant outside of allowed efficiency ranges 11% of the time (e.g., power < 57 MW and heat > 146 MMBTU).

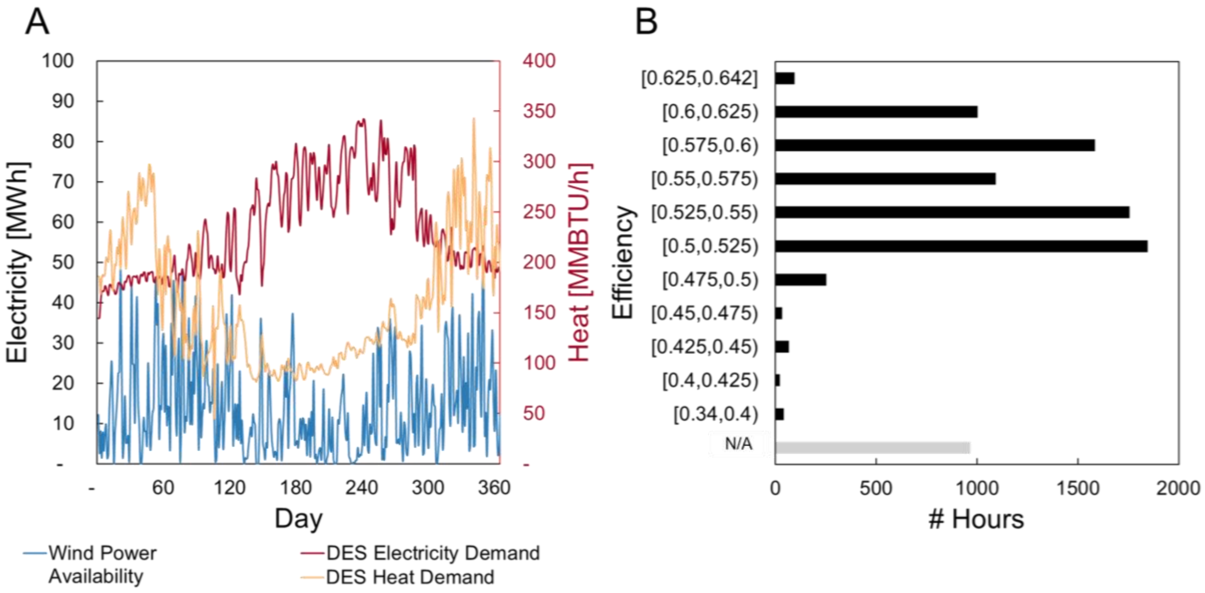

Figure S4. Operation profile of the DES and CHP. (a) Heat and electricity required in the district during the year and the wind power availability and (b) Histogram for CHP efficiency. The light gray bar shows that the CHP did not operate in 11% of the year given the operational rules of the DES.

8. Analyzing alternatives for the base-design project

Table S4. Economic performance and emissions of different alternatives for the base-design project. The only viable project is the PPA Spillage with CCUS. No project profits from H_2 sale, the high integration ones receive important benefits from O_2 savings and carbon prices have mixed effects across alternatives.

**Annual Costs [$ M/y]**	**High SOEC Integration, CCUS**	**High SOEC Integration, no CCUS**	**PPA Spillage, CCUS**	**PPA Spillage, no CCUS**	**External from RE**	**External from RE + CCUS in CHP**	**External from NG (No CCUS)**	**External from NG (with CCUS)**	**External, from NG (with CCUS) + CCUS in CHP**
H_2	0.00	0.00	0.00	0.00	-37.08	-37.08	-9.83	-15.20	-15.20
O_2	1.39	1.39	0.02	0.02	0.00	0.00	0.00	0.00	0.00
CO_2 Capt	12.50	0.00	11.76	0.00	0.00	8.24	0.00	0.00	8.24
Grid	-5.40	0.01	-5.09	0.00	0.00	-3.56	0.00	0.00	-3.56
CHP Var. expense	-0.13	-0.13	-0.01	-0.01	0.57	0.57	0.57	0.57	0.57
Carbon T&S	-4.86	0.00	-4.57	0.00	0.00	-3.20	0.00	0.00	-3.20
Carbon Tax	8.53	-0.76	8.74	0.00	4.15	10.27	-31.03	-12.03	-5.91
NG cost	-1.07	-1.07	0.00	0.00	7.32	7.32	7.32	7.32	7.32
Demand Charge	-1.77	-0.05	-1.70	0.00	0.00	-1.01	0.00	0.00	-1.01
SOEC Stack replacements	-0.60	-0.60	-0.01	-0.01	0.00	0.00	0.00	0.00	0.00
Water	-0.03	-0.03	-0.00	-0.00	0.00	0.00	0.00	0.00	0.00
Fix Expense Electrolyzer operation	-0.01	-0.01	-0.01	-0.01	0.00	0.00	0.00	0.00	0.00
Annualized Capex Electrolyzer	-0.82	-0.82	-0.82	-0.82	0.00	0.00	0.00	0.00	0.00
Annualized Capex Carbon Capture	-7.79	0.00	-7.79	0.00	0.00	-7.79	0.00	0.00	-7.79
**Total Annualized Value [$M/y]**	**-0.06**	**-2.08**	**0.51**	**-0.84**	**-25.03**	**-26.24**	**-32.96**	**-19.34**	**-20.54**
**Aunnual Emission Reductions [k Tonne/y]**	**170.54**	**-15.24**	**174.80**	**0.02**	**83.04**	**205.41**	**-620.56**	**-240.61**	**-118.24**
**NPV [$ M]**	**-0.70**	**-23.87**	**5.83**	**-9.65**	**-287.10**	**-300.94**	**-378.03**	**-221.80**	**-235.64**

9. Results of model complexity analysis

We found large differences between the results of our model, compared to assuming average efficiencies at the CHP. In our model, we used specific thermal and electric efficiencies for each operation point, according to simulations in a turbomachinery software, and we find that using the average efficiencies consistently overestimated fuel consumption. Even using the maximum CHP thermal and electric efficiencies (i.e., 0.675 and 0.415, respectively) in the operation range, we got higher efficiencies of our combined model, suggesting that the combined efficiency is a convex function. We present in

Table S5 results for our model and different combinations of average thermal and electric efficiency.

Table S5 Comparison of results for natural gas combusted in the CHP after the project implementation for the base design. Values above the maximum thermal or electric efficiency of the CHP are shown in red font.

	Annual Natural Gas consumed at CHP		Overall, Second Law Efficiency		Additional Natural Gas Consumed in CHP after SOEC project implemented compared to Baseline	
	[G BTU/y]	Diff. With Detailed %	[–]	Diff. With Detailed %	[G BTU/y]	Diff. With Detailed %
Detailed	4,353	_	0.56	_	241	–
Average- Using the max. thermal and electrical efficiency of the CHP (41.5, 67.5)	4,674	7.4%	0.53	-5.4%	293	21.6%
Simplified (40, 65)	4,828	10.9%	0.51	-8.9%	308	27.8%
Simplified (40, 67.5)	4,828	10.9%	0.52	-7.1%	299	24.1%
Simplified (**42.5,** 67.5)	4,594	5.5%	0.53	-5.4%	289	19.9%
Simplified (**45,** 67.5)	4,468	2.6%	0.55	-1.8%	279	15.8%
Simplified (**42.5, 70**)	4,505	3.5%	0.54	-3.6%	281	16.6%
Simplified (**45, 70**)	4,382	0.7%	0.003	0.0%	271	12.4%

10. Determining the relationship for the levelized costs of O_2 and carbon prices in a specific project

To analyze the relationship between multiple product prices, we can characterize linear relationships between breakeven prices for two different commodities. In Figure 3 in the main manuscript, we show how the High-integration SOEC with CCUS breaks even without requiring carbon tax or revenues from capture at oxygen prices of $3.2. We can represent, with a linear relationship (Eq. *S4*), the O_2 and carbon prices' conditions to make a high SOEC integration project viable.

$$\frac{NPV_{NoRevenues}}{(Life) \times (Discount\ Factor)} = Q_{O_2}(P_{o_2}) + Q_{CO_{2_{reduced}}}(P_{co_2})$$

$$\frac{-257.9M}{20 \times 0.57} = 6.95M[kg]\left(P_{o_2}\left[\frac{\$}{kg}\right]\right) + 170.5k[T]\left(P_{co_2}\left[\frac{\$}{T}\right]\right)$$

$$132.7 - 144.7P_{o_2} = P_{co_2} \qquad Eq.\ S4$$

11. Effect of Wind and Energy Demand Uncertainty

We analyzed the effects of varying energy demand levels and wind availability conditions, finding that, similar to the results presented in the main manuscript, alternatives present distinct robustness profiles. While variability for the *PPA Spillage* alternative remains steady across the studied range (showing low sensitivity to O_2 prices), *Hi-SOEC* projects exhibit large variability to these conditions (as indicated by the spread of the red and orange lines in Figure 4A in the main manuscript). Furthermore, the impact of variability to demand and wind changes along the analyzed O_2 price range with a sweet spot in which minimal variation occurs across scenarios. These sweet spots occur at different O_2 prices depending on the electrolyzer size – i.e., it is located at \$0.7/kg_{O2} for the 10-MW_{in} SOEC and close to \$1.5/kg_{O2} for the base case. From these points, a system operator can infer which markets a particular design may be robust to uncertainty in wind and energy demand.

12. Scenarios for market and operational conditions analyzed in sensitivity analysis

We further analyzed the effects of project decisions with a sensitivity analysis for external parameters by modeling the following cases:

- A scenario where SOEC capex decreases by 50% compared to 2024 values (based on 2030 projections),[16,25–27] achieving costs comparable with optimistic estimates for the cost of first-of-a-kind turnkey project.[28] This scenario represents the option of deferring the project to benefit from substantial learning-by-doing in reference growth scenarios.[16] However, this is only a reference as electrolysis growth projections are often overly optimistic.[29]
- CCUS capital costs variations (+/- 25%). Despite some of the key pre- and post-combustion CCUS processes are considered mature – i.e., Technology Readiness Index of 7 to 9, corresponding to commercial deployment stage –[30] there are few reported capex references, and substantial expected cost reductions for some components.[31] We assumed a CCUS cost of 76% of the capital cost of the natural gas fired power plant, using a factor reported by Rubin et al.[23] However, the same author estimates capex reductions of 20% after achieving large technology deployment (i.e., 100-GW installed capacity).[32] The reference value for a natural gas fired power plant from the Energy Information and Administration's Annual Energy Outlook.[24]
- Increased durability of the SOEC Stack from 20,000 hours to 30,000 hours, a conservative improvement, as durability is expected to increase by a factor of four by 2050.[16]
- Scenarios of high and low heat and electricity demand and wind generation compared to the reference year (+/- 25%).
- High and low natural gas price and retail electricity price (+/- 25%).
- Receive IRA benefits for low-carbon H_2 production and for carbon management systems, receiving rebates of \$0.6/kg of low-carbon H_2 produced and various values for captured carbon: \$17, \$60, and \$85 /tonne of captured CO_2.[13]

13. Symbols

H_2:	Hydrogen
$SOEC$:	Solid Oxide Electrolyzer Cells
HTE:	High Temperature Electrolyzer
GHG:	Green House Gas
DES:	District Energy System
$CCUS$:	Carbon Capture, Utilization, and Storage
CHP:	Combined Heat and Power Plant
O_2:	Oxygen
BaU:	Business as Usual
RE :	Renewable Energy
MFS:	Multifunctional Systems
IRA:	Inflation Reduction Act of 2022
TEA:	Technoeconomic assessment
$LCO -$:	Levelized Cost of (Products)
$LCOH$:	Levelized Cost of Hydrogen
PPA:	Power Purchase Agreement
C_E:	Cost of Electrical Services [$]
S:	Electrolyzer Size [MW_{IN}]
C_{Trans}:	Transmission Charges [$]
R:	Revenues
$Tonne$:	Metric Tonnes
E:	Electricity supplied by the PPA [MWh]
F:	Fuel
Q:	Heat [MMBTU]
K:	Cost Coefficient [$/unit]
P_{Trans}:	Peak Transmission [MW]
P_{Cap}:	Representative Annual Peak Transmission [MW]
D:	Demand
C_f:	Capacity factor (% of installed capacity units)
η:	Efficiency
ΔH:	Total Energy Requirements in Electrolyzer
ΔS:	Heat Requirement of Electrolyzer
ΔG:	Gibbs Energy, Electrical Requirement of Electrolyzer
WH-WM-WL	Wind High, Wind Medium, Wind Low (Scenarios)
DH-DM-DL	Demand High, Demand Medium, Demand Low (Scenarios)

Subscripts:

CHP	Metric or process associated with the Combined Heat and Power plant
CCUS	Carbon Capture, Utilization, and Storage
NG	Natural gas

B	Boiler
Th	Thermal
El	Electrical
t	Superscript associated to period “t” in a specific hour
$Base$	Baseline
$Trans$	Associated to Transmission Charges
Cap	Associated to Capacity Charges
Var	Variable Cost
S_M	Escalation Factor of a Component
W	Wind
Hr	High Resolution (hourly)
Avg	Annual Average

14. References

(1) Sochański, P.; Gładysz, P.; Hasegawa, H. Polygeneration System for Hydrogen-Containing Synthetic Fuel Production: A Techno-Economic and Environmental Assessment. *J. Phys.: Conf. Ser.* **2024**, *2812* (1), 012007. DOI: 10.1088/1742-6596/2812/1/012007.

(2) Safder, U.; Loy-Benitez, J.; Yoo, C. Techno-Economic Assessment of a Novel Integrated Multigeneration System to Synthesize e-Methanol and Green Hydrogen in a Carbon-Neutral Context. *Energy* **2024**, *290*, 130104. DOI: 10.1016/j.energy.2023.130104.

(3) Acen, C.; Bamisile, O.; Adedeji, M.; Cai, D.; Dagbasi, M.; Hu, Y.; Staffell, I. Energy, Exergy, and Exergoeconomic Cost Optimization of Wind-Biomass Multi-Energy Systems Integrated for Hydrogen Production. *J Therm Anal Calorim* **2024**, *149* (16), 8799–8812. DOI: 10.1007/s10973-024-13135-2.

(4) Wang, Q.; Macián-Juan, R.; Yang, M.; Zhang, P.; Liu, X.; Yang, B.; Li, R.; Cheng, H.; Wang, Y.; Fang, S.; Ye, X.; Xiong, W. Thermo-Economic Characteristics and Cost-Influencing Mechanism Analysis of an Advanced Nuclear-Powered Zero-Carbon Hydrogen-Electricity Co-Production System with Sulfur-Iodine Process and Combined Cycle. *International Journal of Hydrogen Energy* **2024**, *78*, 688–702. DOI: 10.1016/j.ijhydene.2024.06.323.

(5) Giwa, T.; Akbari, M.; Kumar, A. Techno-Economic Assessment of an Integrated Biorefinery Producing Bio-Oil, Ethanol, and Hydrogen. *Fuel* **2023**, *332*, 126022. DOI: 10.1016/j.fuel.2022.126022.

(6) Ng, K. S.; Zhang, N.; Sadhukhan, J. Techno-Economic Analysis of Polygeneration Systems with Carbon Capture and Storage and CO2 Reuse. *Chemical Engineering Journal* **2013**, *219*, 96–108. DOI: 10.1016/j.cej.2012.12.082.

(7) Ng, K. S.; Phan, A. N.; Iacovidou, E.; Wan Ab Karim Ghani, W. A. Techno-Economic Assessment of a Novel Integrated System of Mechanical-Biological Treatment and Valorisation of Residual Municipal Solid Waste into Hydrogen: A Case Study in the UK. *Journal of Cleaner Production* **2021**, *298*, 126706. DOI: 10.1016/j.jclepro.2021.126706.

(8) Cao, Y.; Dhahad, H. A.; Togun, H.; Anqi, A. E.; Farouk, N.; Farhang, B. A Novel Hybrid Biomass-Solar Driven Triple Combined Power Cycle Integrated with Hydrogen Production: Multi-Objective Optimization Based on Power Cost and CO2 Emission. *Energy Conversion and Management* **2021**, *234*, 113910. DOI: 10.1016/j.enconman.2021.113910.

(9) Jiang, P.; Berrouk, A. S.; Dara, S. Biomass Gasification Integrated with Chemical Looping System for Hydrogen and Power. Coproduction Process – Thermodynamic and Techno-Economic Assessment. *Chemical Engineering & Technology* **2019**, *42* (5), 1153–1168. DOI: 10.1002/ceat.201900130.

(10) Szima, S.; Cormos, C.-C. Techno – Economic Assessment of Flexible Decarbonized Hydrogen and Power Co-Production Based on Natural Gas Dry Reforming. *International Journal of Hydrogen Energy* **2019**, *44* (60), 31712–31723. DOI: 10.1016/j.ijhydene.2019.10.115.

(11) Spallina, V.; Motamedi, G.; Gallucci, F.; van Sint Annaland, M. Techno-Economic Assessment of an Integrated High Pressure Chemical-Looping Process with Packed-Bed Reactors in Large Scale Hydrogen and Methanol Production. *International Journal of Greenhouse Gas Control* **2019**, *88*, 71–84. DOI: 10.1016/j.ijggc.2019.05.026.

(12) Cormos, C.-C. Biomass Direct Chemical Looping for Hydrogen and Power Co-Production: Process Configuration, Simulation, Thermal Integration and Techno-Economic Assessment. *Fuel Processing Technology* **2015**, *137*, 16–23. DOI: 10.1016/j.fuproc.2015.04.001.

(13) 117th U.S. Congress (2021-2022). *H.R.5376 - 117th U.S. Congress (2021-2022): Inflation Reduction Act of 2022*; 2022. https://www.congress.gov/bill/117th-congress/house-bill/5376/text (accessed 2025-02-16).

(14) Wilkinson, J.; Mays, T.; McManus, M. Review and Meta-Analysis of Recent Life Cycle Assessments of Hydrogen Production. *Cleaner Environmental Systems* **2023**, *9*, 100116. DOI: 10.1016/j.cesys.2023.100116.
(15) DOE. *Energy Earthshots Initiative> Hydrogen Shot | Department of Energy*. Energy Earthshots Initiative. https://www.energy.gov/policy/energy-earthshots-initiative (accessed 2023-07-12).
(16) IRENA. *Green Hydrogen Cost Reduction: Scaling up Electrolysers to Meet the 1.5^{0}C Climate Goal*; International Renewable Energy Agency: Abu Dhabi, 2020. https://www.irena.org/publications/2020/Dec/Green-hydrogen-cost-reduction (accessed 2023-08-22).
(17) NETL. *Comparison of Commercial, State-of-the-Art, Fossil-Based Hydrogen Production Technologies*; DOE/NETL2022/3241; 2022. https://netl.doe.gov/node/11701 (accessed 2022-09-19).
(18) Al-Qahtani, A.; Parkinson, B.; Hellgardt, K.; Shah, N.; Guillen-Gosalbez, G. Uncovering the True Cost of Hydrogen Production Routes Using Life Cycle Monetisation. *Applied Energy* **2021**, *281*, 115958. DOI: 10.1016/j.apenergy.2020.115958.
(19) Maggio, G.; Squadrito, G.; Nicita, A. Hydrogen and Medical Oxygen by Renewable Energy Based Electrolysis: A Green and Economically Viable Route. *Applied Energy* **2022**, *306*, 117993. DOI: 10.1016/j.apenergy.2021.117993.
(20) Wang, P.; Deng, X.; Zhou, H.; Yu, S. Estimates of the Social Cost of Carbon: A Review Based on Meta-Analysis. *Journal of Cleaner Production* **2019**, *209*, 1494–1507. DOI: 10.1016/j.jclepro.2018.11.058.
(21) Pindyck, R. S. The Social Cost of Carbon Revisited. *Journal of Environmental Economics and Management* **2019**, *94*, 140–160. DOI: 10.1016/j.jeem.2019.02.003.
(22) NETL. *Cost and Performance Baseline for Fossil Energy Plants VOL 1: Bituminous Coal and Natural Gas to Electricity*; NETL-PUB-22638; National Energy Technology Laboratory (NETL), Pittsburgh, PA, 2019. https://netl.doe.gov/energy-analysis/details?id=e818549c-a565-4cbc-94db-442a1c2a70a9 (accessed 2024-04-28).
(23) Rubin, E. S.; Davison, J. E.; Herzog, H. J. The Cost of CO2 Capture and Storage. *International Journal of Greenhouse Gas Control* **2015**, *40*, 378–400. DOI: 10.1016/j.ijggc.2015.05.018.
(24) EIA. *Annual Energy Outlook 2023*; 2023. https://www.eia.gov/outlooks/aeo/index.php (accessed 2024-11-22).
(25) Böhm, H.; Zauner, A.; Rosenfeld, D. C.; Tichler, R. Projecting Cost Development for Future Large-Scale Power-to-Gas Implementations by Scaling Effects. *Applied Energy* **2020**, *264*, 114780. DOI: 10.1016/j.apenergy.2020.114780.
(26) Böhm, H.; Moser, S.; Puschnigg, S.; Zauner, A. Power-to-Hydrogen & District Heating: Technology-Based and Infrastructure-Oriented Analysis of (Future) Sector Coupling Potentials. *International Journal of Hydrogen Energy* **2021**, *46* (63), 31938–31951. DOI: 10.1016/j.ijhydene.2021.06.233.
(27) Ni, M.; Leung, M. K. H.; Leung, D. Y. C. Technological Development of Hydrogen Production by Solid Oxide Electrolyzer Cell (SOEC). *International Journal of Hydrogen Energy* **2008**, *33* (9), 2337–2354. https://doi.org/10.1016/j.ijhydene.2008.02.048.
(28) Anghilante, R.; Colomar, D.; Brisse, A.; Marrony, M. Bottom-up Cost Evaluation of SOEC Systems in the Range of 10–100 MW. *International Journal of Hydrogen Energy* **2018**, *43* (45), 20309–20322. DOI: 10.1016/j.ijhydene.2018.08.161.
(29) Odenweller, A.; Ueckerdt, F. The Green Hydrogen Ambition and Implementation Gap. *Nat Energy* **2025**, 1–14. DOI: 10.1038/s41560-024-01684-7.
(30) Dziejarski, B.; Krzyżyńska, R.; Andersson, K. Current Status of Carbon Capture, Utilization, and Storage Technologies in the Global Economy: A Survey of Technical Assessment. *Fuel* **2023**, *342*, 127776. DOI: 10.1016/j.fuel.2023.127776.

(31) Yusuf, M.; Ibrahim, H. A Comprehensive Review on Recent Trends in Carbon Capture, Utilization, and Storage Techniques. *Journal of Environmental Chemical Engineering* **2023**, *11* (6), 111393. DOI: 10.1016/j.jece.2023.111393.
(32) Rubin, E. S.; Yeh, S.; Antes, M.; Berkenpas, M.; Davison, J. Use of Experience Curves to Estimate the Future Cost of Power Plants with CO2 Capture. *International Journal of Greenhouse Gas Control* **2007**, *1* (2), 188–197. DOI: 10.1016/S1750-5836(07)00016-3.
